# hPMSCs inhibit the expression of PD-1 in CD4^+^IL-10^+^ T cells and mitigate liver damage in a GVHD mouse model by regulating the crosstalk between Nrf2 and NF-κB signaling pathway

**DOI:** 10.1186/s13287-021-02407-5

**Published:** 2021-06-29

**Authors:** Aiping Zhang, Jiashen Zhang, Xiaohua Li, Hengchao Zhang, Yanlian Xiong, Zhuoya Wang, Nannan Zhao, Feifei Wang, Xiying Luan

**Affiliations:** 1 Department of Immunology, Binzhou Medical University, Yantai, Shandong Province 264003 People’s Republic of China; 2 Department of Component, Yantai Central Blood Station, Yantai, Shandong Province 264003 People’s Republic of China; 3 Department of Anatomy, Binzhou Medical University, Yantai, Shandong Province 264003 People’s Republic of China; 4 Department of Anesthesiology, Yantai Affiliated Hospital of Binzhou Medical University, Shandong Province 264003 Yantai, People’s Republic of China

**Keywords:** Human placenta-derived mesenchymal stromal cells, Programmed death-1, CD4^+^IL-10^+^ T cells, Nuclear factor-E2-related factor 2, Nuclear factor kappa-B, Graft-versus-host disease

## Abstract

**Background:**

The activation of T cells and imbalanced redox metabolism enhances the development of graft-versus-host disease (GVHD). Human placenta-derived mesenchymal stromal cells (hPMSCs) can improve GVHD through regulating T cell responses. However, whether hPMSCs balance the redox metabolism of CD4^+^IL-10^+^ T cells and liver tissue and alleviate GVHD remains unclear. This study aimed to investigate the effect of hPMSC-mediated treatment of GVHD associated with CD4^+^IL-10^+^ T cell generation via control of redox metabolism and PD-1 expression and whether the Nrf2 and NF-κB signaling pathways were both involved in the process.

**Methods:**

A GVHD mouse model was induced using 6–8-week-old C57BL/6 and Balb/c mice, which were treated with hPMSCs. In order to observe whether hPMSCs affect the generation of CD4^+^IL-10^+^ T cells via control of redox metabolism and PD-1 expression, a CD4^+^IL-10^+^ T cell culture system was induced using human naive CD4^+^ T cells. The percentage of CD4^+^IL-10^+^ T cells and their PD-1 expression levels were determined in vivo and in vitro using flow cytometry, and Nrf2, HO-1, NQO1, GCLC, GCLM, and NF-κB levels were determined by western blotting, qRT-PCR, and immunofluorescence, respectively. Hematoxylin-eosin, Masson’s trichrome, and periodic acid-Schiff staining methods were employed to analyze the changes in hepatic tissue.

**Results:**

A decreased activity of superoxide dismutase (SOD) and a proportion of CD4^+^IL-10^+^ T cells with increased PD-1 expression were observed in GVHD patients and the mouse model. Treatment with hPMSCs increased SOD activity and GCL and GSH levels in the GVHD mouse model. The percentage of CD4^+^IL-10^+^ T cells with decreased PD-1 expression, as well as Nrf2, HO-1, NQO1, GCLC, and GCLM levels, both in the GVHD mouse model and in the process of CD4^+^IL-10^+^ T cell generation, were also increased, but NF-κB phosphorylation and nuclear translocation were inhibited after treatment with hPMSCs, which was accompanied by improvement of hepatic histopathological changes.

**Conclusions:**

The findings suggested that hPMSC-mediated redox metabolism balance and decreased PD-1 expression in CD4^+^IL-10^+^ T cells were achieved by controlling the crosstalk between Nrf2 and NF-κB, which further provided evidence for the application of hPMSC-mediated treatment of GVHD.

## Background

The tissue damage caused by donor T cell activation, imbalanced proportion of T cell subsets, and oxidative stress after allogeneic hematopoietic stem cell transplantation (allo-HSCT) contributes to the development of graft-versus-host disease (GVHD), which is also the main obstacle to the success of allo-HSCT treatment. It was reported that human placenta-derived mesenchymal stromal cells (hPMSCs) could improve GVHD by increasing the proportion of Th2 and CD4^+^CD25^+^FoxP3^+^ T cells via PD-L1 expressed on the hPMSC surface and by subsequently alleviating liver damage [1, 2]. Recently, in addition to FoxP3^+^ T cells, IL-10-producing CD4^+^ T (CD4^+^IL-10^+^ T) cells have also attracted considerable attention in GVHD cell therapy because of the exertion of their negative immunomodulatory effects. It was indicated that endogenous and exogenous IL-10 could directly or indirectly inhibit the production of pro-inflammatory factors, such as IL-1β, and the proliferation of T cells, thus blocking elicitation of the immune response and reducing inflammatory damage in a severe colitis mouse model [3]. Subsequently, CD4^+^IL-10^+^ T cells were shown to exert immunosuppressive effects in various disease models, such as inflammatory bowel disease (IBD) and experimental autoimmune encephalomyelitis (EAE) [4, 5]. Our previous results showed that hPMSCs could alleviate GVHD symptoms by maintaining the balance of CD4^+^IL-10^+^ T cells and Th17 subsets [6]; however, the specific mechanism by which hPMSCs regulate the generation of CD4^+^IL-10^+^ T cells remains unclear.

Programmed death-1 (PD-1), as a negative costimulatory molecule expressed on the surface of activated T cells, can induce T cell apoptosis by binding to its ligand PD-L1/2 or by upregulating the expression of suppressor genes. It is evident that the percentage of PD-1^+^ T cells is increased during GVHD development, and treatment with PD-1 as a target molecule can effectively improve the symptoms of GVHD, which indicates that PD-1 expression plays an important role in the pathogenesis and treatment of GVHD [7]. Interestingly, PD-1 is also expressed on the surface of CD4^+^IL-10^+^ T cells [8] and the proportion of CD4^+^IL-10^+^ T cells is significantly decreased in GVHD patients and in mouse models [9]. However, it remains elusive whether the decreased number of CD4^+^IL-10^+^ T cells is associated with the expression of PD-1.

Several studies have confirmed that the activation and proliferation of T cells during the development of GVHD is accompanied by a marked increase in energy metabolism. During this process, the levels of reactive oxygen species (ROS), carbonylation, and malondialdehyde (MDA) increase remarkably, which lead to the development of cell and liver damage [10, 11]. Enzymes and nonenzymatic antioxidants, such as glutathione (GSH) and superoxide dismutase (SOD), play important roles in the maintenance of normal physiological functions. When GVHD occurs, the imbalance in redox metabolism markedly exacerbates the inflammatory response elicited and tissue damage caused therein; however, the GSH redox status contributes to the mitigation of target tissue damage in GVHD [12]. GSH is the main nonenzymatic antioxidant and plays a key role in maintaining ROS homeostasis during T cell activation and in regulating the metabolic reprogramming of Myc-dependent T cells, while GSH can also reduce the production of Th17 cells [13, 14]. Similarly, a previous study has shown that the ratio of GSH/GSSG is reduced in GVHD patients and a GVHD mouse model, and the level of ROS in T cells is positively correlated with PD-1 expression. Additionally, hPMSCs can mitigate inflammatory responses via reduction of the expression of PD-1 in T cells by increasing the levels of GSH and GST in T cells during GVHD [7]. However, whether hPMSCs can promote the production of CD4^+^IL-10^+^ T cells by regulating PD-1 expression and reduce the inflammatory response by interfering with redox metabolism during GVHD is unclear.

Nuclear factor-E2-related factor 2 (Nrf2), a key transcription factor, can resist the development of oxidative damage in cells, influence the balance of redox metabolism and signaling, and promote the expression of a series of genes that confer protection against oxidative stress, such as NAD (P) H: quinone oxidoreductase 1 (NQO1), heme oxygenase 1 (HO-1), glutamate-cysteine ligase regulatory subunit (GCLM), and glutamate-cysteine ligase catalytic subunit (GCLC). The expression of these genes can reduce oxidative and inflammatory damage and enhance antioxidant capacity in the body [15, 16]. Moreover, Nrf2 expression is also involved in the differentiation of Th2 cells and promotes the transcription of IL-10 in T cells [17]. Piantadosi et al. have shown that the deletion of Nrf2 or the related gene HO-1 leads to a decrease in IL-10 levels in macrophages [18]. However, whether hPMSCs can regulate the generation of CD4^+^IL-10^+^ T cells via the Nrf2 signaling pathway, thereby attenuating GVHD symptoms, remains unknown. Additionally, the activation of Nrf2 was strengthened via the downregulation of NF-κB activation, which promoted the binding of Nrf2 and CREB-binding protein (CBP) and further regulated the expression of downstream target genes [19]. However, whether the crosstalk between NF-κB and Nrf2 is also involved in the process by which hPMSCs improve GVHD symptoms or induce the generation of CD4^+^IL-10^+^ T cells warrants exploration.

In this study, an allogeneic GVHD mouse model was induced to explore the effect of hPMSC-mediated regulation of PD-1 expression on CD4^+^IL-10^+^ T cells, redox metabolism, and liver damage. Furthermore, a CD4^+^IL-10^+^ T cell subset differentiation culture system was established in vitro to investigate whether hPMSCs could inhibit the expression of PD-1 by regulating the crosstalk between NF-κB and Nrf2 and, in turn, induce the generation of CD4^+^IL-10^+^ T cells. These results may provide a new theoretical basis for the treatment of GVHD with hPMSCs.

## Materials and methods

### Patients

GVHD patients and healthy donors from Yuhuangding Hospital and the Central Blood Bank in Yantai, China, respectively, signed informed consent forms. All patients met the GVHD diagnostic criteria, and the experiments were approved by the Yuhuangding hospital ethics committee. Patient information is shown in Table 1.

**Table 1:** Patient information

GVHD patient no.	1	2	3	4	5	6	7
Age (y)	30–39	20–29	20–29	50–59	40–49	50–59	0–9
Sex	1	2	1	1	1	1	1
Diagnosis	ALL	AML	AA	AML	ALL	ALL	AA
Stem cell source	HSC and PBSC	HSC and PBSC	HSC and PBSC	HSC and PBSC	HSC and PBSC	HSC and PBSC	HSC and PBSC
GVHD prophylaxis	Tacrolimus	Tacrolimus and cyclosporine A	Cyclosporine A	Cyclosporine A and mycophenolic acid and methotrexate	Cyclosporine A and mycophenolic acid and methotrexate	Cyclosporine A and mycophenolic acid	Cyclosporine A
Match	MFD	MFD	MFD	MFD	MFD	MFD	MFD
Concomitant drug therapy	Tacrolimus	Tacrolimus	Cyclosporine A	Methylprednisolone and cyclosporine A	Cyclosporine A	Cyclosporine A	Cyclosporine A

*ALL* acute lymphoblastic leukemia, *AML* acute myelocytic leukemia, *AA* aplastic anemia, *HSC* hematopoietic stem cell, *PBMC* peripheral blood stem cell, *MFD* matched familiar donor

### Mice

Six- to 8-week-old male C57BL/6 mice and female Balb/c mice were purchased from Ji’nan Pengyue Laboratory Animal Breeding Company (Ji’nan, China). The mice were housed in a controlled environment (12-h light/12-h dark photoperiod, 22°C ± 1°C, 60% ± 10% relative humidity). All husbandry and experimental contact with the mice were maintained under pathogen-free conditions. All protocols and experimental procedures were approved by the Ethics Committee of Binzhou Medical University Yantai, China.

### Isolation, culture, and identification of hPMSCs

Based on previously described methods [20], placental tissue samples were collected from healthy-term pregnant women from the Affiliated Yantai Hospital of Binzhou Medical University, Yantai, China, who signed informed consent forms. Washed and crushed placentas were then incubated with gentle shaking at 37°C in 0.1% collagenase IV (Gibco, Grand Island, USA) for 30 min. Low-glucose Dulbecco’s modified Eagle medium (DMEM) supplemented with 10% fetal bovine serum (FBS; Gibco, Grand Island, USA) was used to terminate the collagenase-mediated digestion. The cell suspension was filtered through a 100-mm nylon membrane, centrifuged at 524*g* for 10 min, resuspended in low-glucose DMEM supplemented with 10% FBS (Gibco, Grand Island, USA), 100 U/mL penicillin G, and 100 U/mL streptomycin sulfate, and then cultured at 37°C in a humidified 5% CO_2_ atmosphere. The medium was replaced either once or twice each week with a fresh medium. After three passages, the isolated cells were analyzed by microscopy for morphological analysis, by flow cytometry (FCM) for immunophenotype analysis, and by adipogenic and osteogenic staining for multidirectional differentiation analysis. The project was approved by the Ethics Committee of Binzhou Medical University, Yantai, China.

### Induction of the GVHD mouse model and treatment

The GVHD mouse model was induced as per previously described methods [7]. The female Balb/c recipients were irradiated using 8 Gy of X-ray irradiation (2.19 Gy/min), at intervals of 4–6 h. Donor bone marrow cells (5 × 10^6^) and splenic mononuclear cells (5 × 10^6^) obtained from male C57BL/6J mice were resuspended in 50 μL of PBS and transplanted into irradiated female Balb/c mice via tail vein injection. Five days after transplantation, hPMSCs (1 × 10^6^) suspended in 50 μL of PBS or PBS were administered via the tail vein. The mice were sacrificed, and target organs were harvested from each group for performance of specific experiments at 0 and 7 days after the transplantation of hPMSCs or administration of PBS. Additionally, body weights were measured, and GVHD scores were assessed using a scoring system as per previously described methods [20].

### Differentiation of human CD4^+^IL-10^+^ T cells

Human CD4^+^IL-10^+^ T cells were induced in vitro as per previously described protocols [21]. Human peripheral blood mononuclear cells (PBMCs) were obtained from healthy donor blood samples (Central Blood Bank in Yantai, China) using gradient separation with Ficoll-Hypaque (1.077 g/mL; Solarbio, Beijing, China). PBMCs were cultured using the RPMI-1640 medium (HyClone, Boston, MA) and incubated at 37°C, and naive CD4^+^ T cells were separated using a naive CD4^+^ T cell isolation kit (human; Miltenyi Biotec, San Diego, CA) according to the manufacturer’s instructions.

Naive CD4^+^ T cells were treated with or without the Nrf2 inhibitor-ML385 (10 μM, MCE, NJ, USA) for 12 h, and then naive CD4^+^ T cells were cultured with or without hPMSCs. Anti-human CD3ε and anti-human CD28 mAb (1 μg/mL, Life Technologies, Waltham, MA, USA), recombinant human (rh) IL-2 (200 U/mL, Proteintech, Wuhan, China), IL-10 (100 U/mL, Proteintech), IL-27 (63 U/μL, Proteintech), and IFN-α2b (2.09 U/μL, PBL Assay Science, NJ, USA) were added to the coculture system. After 3 days, the cells were stimulated using PMA/ionomycin (1.25 μg/mL, 0.25 mg/mL, MultiSciences, Hangzhou, China) and BFA/monensin (0.75 mg/mL, 0.25 mg/mL, MultiSciences) for 5 h and subjected to surface staining and intracellular staining procedures.

### Western blotting

The protein levels of Nrf2, HO-1, NQO1, GCLC, GCLM, NF-κB, and I-κB and the phosphorylation status of NF-κB and I-κB (p-NF-κB and p-I-κB) respectively in the liver, spleen, and mononuclear cells of GVHD mice and human CD4^+^IL-10^+^ T cells were analyzed by performing western blotting. The tissue and cellular protein extracts were subjected to SDS-PAGE and electrophoresed proteins were then transferred onto PVDF membranes. Antibodies for Nrf2, I-κB, p-NF-κB, p-I-κB, NQO1 (Abcam, Cambridge, UK), HO-1, NF-κB (Cell Signaling Technology, MA, USA), GCLC, and GCLM (Proteintech, Wuhan, China) were added to the membranes and the membranes were incubated overnight at 4°C. The membranes were then washed 4 times, goat anti-rabbit and goat anti-mouse secondary antibodies (Proteintech, Wuhan, China) were added, and samples were incubated for 2 h at room temperature. A western blot imager was used to observe the protein bands after the blots were further washed and developed using an ECL substrate (Beyotime, Nanjing, China).

### Flow cytometry (FCM)

The percentage of CD4^+^IL-10^+^ T cells in PBMCs and PD-1 expression in samples obtained from GVHD patients and healthy donors and in the mononuclear cells in the liver and spleen of the GVHD mouse model were evaluated using the FACSCanto II flow cytometer (BD Biosciences). Briefly, cells were incubated with antibodies against surface markers at 4°C for 25 min in the dark. To perform intracellular staining, the cells were fixed, permeabilized, and stained with fluorescently labeled antibodies for an additional 25 min at 4°C in the dark.

The following antibodies were used: anti-mouse CD3-PerCP-Cy5.5 (BD Biosciences, CA, USA), anti-mouse CD4-APC (BD Biosciences), anti-mouse IL-10-FITC (BD Biosciences), anti-mouse PD-1-PE (BD Biosciences), anti-human CD4-APC (BD Biosciences), anti-human IL-10-FITC (BD Biosciences), and anti-human PD-1-PE (BD Biosciences). An APC Annexin V apoptosis detection kit with 7-AAD (BioLegend, San Diego, CA) was also used.

### Quantitative reverse-transcription PCR

Total RNA was extracted using the TRIzol reagent (Invitrogen, CA, USA) and reverse-transcribed into complementary DNA (cDNA) using the Revert Aid First Strand cDNA Synthesis Kit (Thermo Scientific, CA, USA); further, the Nrf2, NQO1, HO-1, GCLC, and GCLM mRNA levels were analyzed by real-time quantitative polymerase chain reaction (RT-qPCR) using the Thermo Scientific DyNAmo ColorFlash SYBR Green qPCR kit (Thermo Scientific, CA, USA) according to the manufacturer’s instructions. Transcript levels were determined using the comparative critical threshold (Ct) method with normalization to the GAPDH gene expression based on the 2^−ΔΔCt^ calculation. Primer sequences were synthesized and provided by Sangon (Shanghai, China).

Additional primers used were as follows:

**Table:** 

Gene name	Primer sequence
*Nrf2*	Forward primer: 5′-TCCAAGTCCAGAAGCCAAACTGAC-3′
	Reverse primer: 5′-GGAGAGGATGCTGCTGAAGGAATC-3′
*HO-1*	Forward primer: 5′-CCTCCCTGTACCACATCTATGT-3′
	Reverse primer: 5′-GCTCTTCTGGGAAGTAGACAG-3′
*NQO1*	Forward primer: 5′-GTCGGCAGAAGAGCACTGATCG-3′
	Reverse primer: 5′-ACTCCACCACCTCCCATCCTTTC-3′
*GCLC*	Forward primer: 5′-TGTCCGAGTTCAATACAGTTGA-3′
	Reverse primer: 5′-ACAGCCTAATCTGGGAAATGAA-3′
*GCLM*	Forward primer: 5′-GGGCACAGGTAAAACCAAATAG-3′
	Reverse primer: 5′-TTTTCACAATGACCGAATACCG-3′

### Immunofluorescence

To perform the Nrf2 and NF-κB nuclear translocation experiments, the cells were fixed with 4% paraformaldehyde, washed, and blocked for 2 h using 1% bovine serum albumin (Solarbio, Beijing, China), 10% normal goat serum, and 0.3% Triton X-100 (Solarbio, Beijing, China). Primary antibodies were added and cells were incubated at 4°C overnight. Goat anti-mouse IgG (DyLight 549) and goat anti-rabbit IgG fluorescently labeled secondary antibodies (DyLight 488) were then added, and samples were incubated at room temperature for 2 h. Finally, 4,6-diamidino-2-phenylindole (DAPI) (Cell Signaling Technology, MA, USA) was used and added as a nuclear counterstain and samples were incubated at room temperature for 2 min. The images were acquired after completion of staining experiments by using a fluorescence microscope (Zeiss LSM880 with Airyscan), and the mean fluorescence intensity values were analyzed using the ZEN software. The fluorochromes used included DAPI, red fluorescent protein, and green fluorescent protein.

### Determination of redox metabolism

The activity of GCL and SOD (Beyotime, Nanjing, China) and the concentrations of MDA, GSH, GSSG (Beyotime, Nanjing, China) and carbonyl (Sigma, St Louis, MO, USA) in tissues were quantified according to the manufacturer’s instructions.

### Hematoxylin-eosin (H&E), Masson’s trichrome, and periodic acid-Schiff (PAS) staining

The liver and skin samples were harvested, washed, fixed, and paraffin-embedded as per routine procedures. To perform examination of the organs, 5-μm sections were stained using H&E for histological analysis. For direct visualization of liver damage, Masson’s trichrome staining was performed using a Masson’s trichrome staining kit (Solarbio, Beijing, China) and PAS staining was performed using a periodic acid-Schiff/PAS Stain Kit (Solarbio, Beijing, China).

### Statistical analysis

Data analyses were performed using the SPSS 17.0 software. The data have been presented as mean ± standard deviation (SD), and all analyses were performed either using unpaired Student’s *t*-tests or one-way ANOVA with least significant difference (LSD) tests for multiple comparisons. A *P* value < 0.05 was considered statistically significant.

## Results

### Phenotypic characteristics and differentiation of hPMSCs

These characteristics of isolated cells were consistent with those reported in our previous studies [2, 20]. Briefly, the isolated cells exhibited a typical fibroblastic morphology and could be differentiated to adipocytes and osteocytes, which were identified by performing oil red O and Alizarin red staining procedures, respectively. The FCM results showed that the isolated cells expressed CD73, CD90, and CD105 but not CD34, CD14, HLA-DR, or CD19.

### hPMSCs inhibit the expression of PD-1 in CD4^+^IL-10^+^ T cells during the development of GVHD

**Fig. 1:**
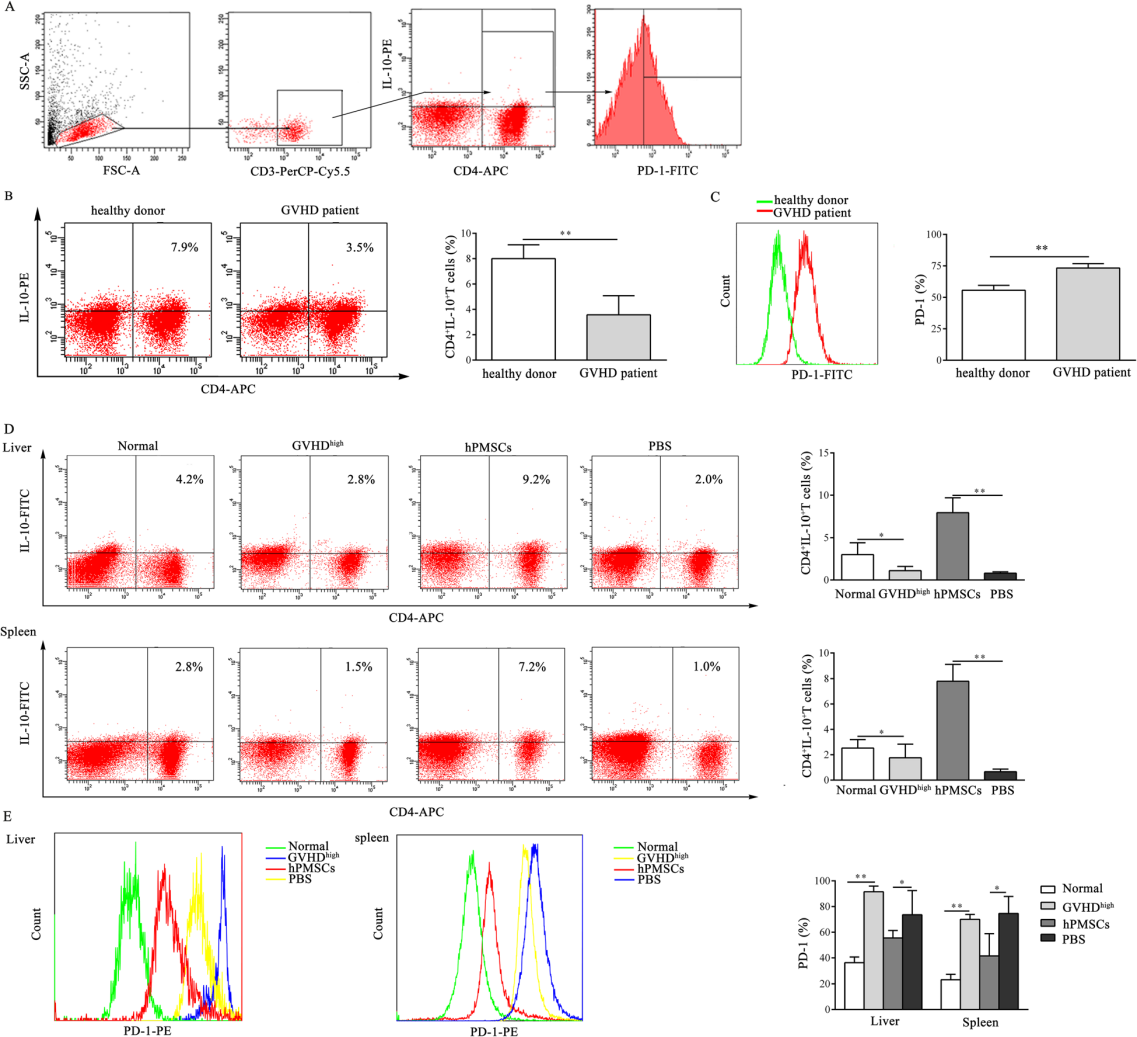
hPMSCs inhibit the expression of PD-1 in CD4^+^IL-10^+^ T cells during the development of GVHD. **a** Gating strategy. Forward scatter (FSC) and side scatter (SSC) gating were used to discriminate viable cells from cell debris. Within the lymphocyte gate, in cells obtained from GVHD patients and the GVHD mouse model, CD3 was used as a T cell marker, CD4^+^IL-10^+^ T cells were defined as those positive for CD4 and IL-10, and PD-1 expression in CD4^+^IL-10^+^ T cells was determined. **b**, **c** Dot plots of a representative experiment of CD4^+^IL-10^+^ T cells and the percentage of CD4^+^IL-10^+^ T cells and the statistical evaluation of the level of PD-1 in CD4^+^IL-10^+^ T cells obtained from GVHD patients and healthy donors (*n* = 7). **d**, **e** Representative dot plots and the percentage of CD4^+^IL-10^+^ T cells and the levels of PD-1 in CD4^+^IL-10^+^ T cells in the spleen and liver of GVHD mice in different groups (*n* = 5). The results were obtained from three independent experiments, *P < 0.05, **P < 0.01

**Fig. 2:**
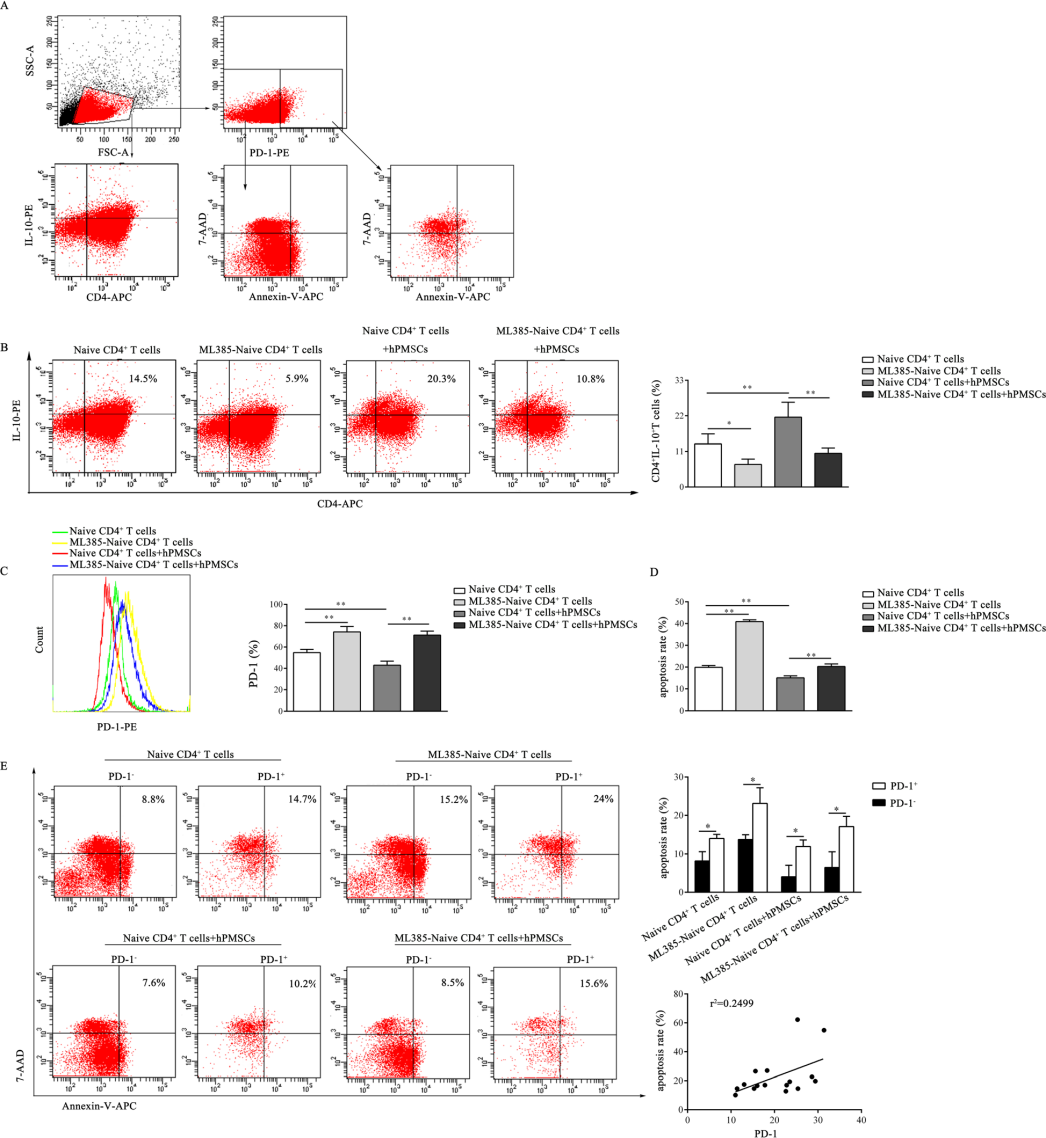
hPMSCs inhibit the expression of PD-1 and the apoptosis of CD4^+^IL-10^+^ T cells in vitro. **a** Gating strategy. Forward scatter (FSC) and side scatter (SSC) gating were used to discriminate viable cells from cell debris. In vitro, the CD4^+^IL-10^+^ T cells were defined as those positive for CD4 and IL-10, and PD-1^+^ cells were defined as those positive for PD-1. Furthermore, the apoptosis rate in PD-1^+^ and PD-1^−^ cells was determined. **b**, **c** Representative dot plots of CD4^+^IL-10^+^ T cells and the percentage of CD4^+^IL-10^+^ T cells and the statistical evaluation of the levels of PD-1 in CD4^+^IL-10^+^ T cells from different groups of CD4^+^IL-10^+^ T cells. **d** The statistical evaluation of the apoptosis in different groups of CD4^+^IL-10^+^ T cells. **e** Dot plots from a representative experiment showing the apoptosis rate of PD-1^+^ and PD-1^−^ cells and the correlation analysis of PD-1 expression and the apoptosis rate. The results were obtained from three independent experiments, *P < 0.05, **P < 0.01

To analyze the effect of hPMSCs on the expression of PD-1 in CD4^+^IL-10^+^ T cells, extracellular PD-1 in gated CD4^+^IL-10^+^ T cells was detected by FCM (Figs. 1a and 2a). The results showed that the proportion of CD4^+^IL-10^+^ T cells decreased in GVHD patients and GVHD^high^ mice (patients: *P* < 0.01, mouse model: *P*<0.05; Fig. 1b, d), similar to the findings of our previous reports [9]. We further observed that PD-1 expression on the surface of CD4^+^IL-10^+^ T cells in both GVHD patients and in the liver and spleen in GVHD^high^ mice was significantly higher than that in healthy donors and the normal mice group (*P* < 0.01, Fig. 1c, e). However, treatment with hPMSCs significantly downregulated the PD-1 expression on the surface of CD4^+^IL-10^+^ T cells in comparison with that of the PBS group (*P* < 0.05, Fig. 1e) and markedly increased the proportion of CD4^+^IL-10^+^ T cells in the liver and spleen in the GVHD mouse model (*P* < 0.01, Fig. 1d).

Considering that the expression of PD-1 in CD4^+^ T cells was positively correlated with ROS levels [7], hPMSCs reduced the ROS levels in T cells by demonstrating the disordered metabolism of GSH, which is downstream of Nrf2. Thus, the effect of Nrf2 was analyzed during the differentiation of CD4^+^IL-10^+^ T cells with or without hPMSCs or by blocking Nrf2 with the Nrf2 inhibitor-ML385. As shown in Fig. 2b, in the presence of hPMSCs, the proportion of CD4^+^IL-10^+^ T cells was significantly increased compared to that of the group lacking hPMSCs (*P* < 0.01); however, the expression of PD-1 on CD4^+^IL-10^+^ T cells was significantly lower than that in the group without hPMSCs (*P* < 0.01, Fig. 2c). After naive CD4^+^ T cells were pretreated with ML385, the proportion of CD4^+^IL-10^+^ T cells was significantly reduced (*P* < 0.05, Fig. 2b), but the expression of PD-1 in the ML385-pretreated group was increased (*P* < 0.01, Fig. 2c) compared to that in the non-pretreatment group. Moreover, when naive CD4^+^ T cells were pretreated with ML385 and cocultured with hPMSCs, the proportion of CD4^+^IL-10^+^ T cells decreased significantly (*P* < 0.01, Fig. 2b) and the expression of PD-1 increased markedly (*P* < 0.01, Fig. 2c) compared to that of the non-pretreated naive CD4^+^ T cells cocultured with hPMSCs.

The expression of PD-1 on T cells can induce T cell apoptosis and reduce the number of T cells. To confirm whether hPMSCs could affect the proportion of CD4^+^IL-10^+^ T cells by regulating CD4^+^IL-10^+^ T cell apoptosis via PD-1 expression, the effect of hPMSCs on the apoptosis of CD4^+^IL-10^+^ T cells was analyzed by FCM (Fig. 2a). The results showed that after naive CD4^+^ T cells were pretreated with ML385, CD4^+^IL-10^+^ T cell apoptosis was significantly increased (*P* < 0.01, Fig. 2d) compared to that of the non-pretreated group. After treatment with hPMSCs, CD4^+^IL-10^+^, T cell apoptosis was significantly decreased (*P* < 0.05, Fig. 2d). Further correlation analysis showed that there was a significant positive correlation between the apoptosis rate and the expression of PD-1 in CD4^+^IL-10^+^ T cells (*P* < 0.05, Fig. 2e).

### hPMSCs improve redox metabolism and alleviate the symptoms of GVHD

**Fig. 3:**
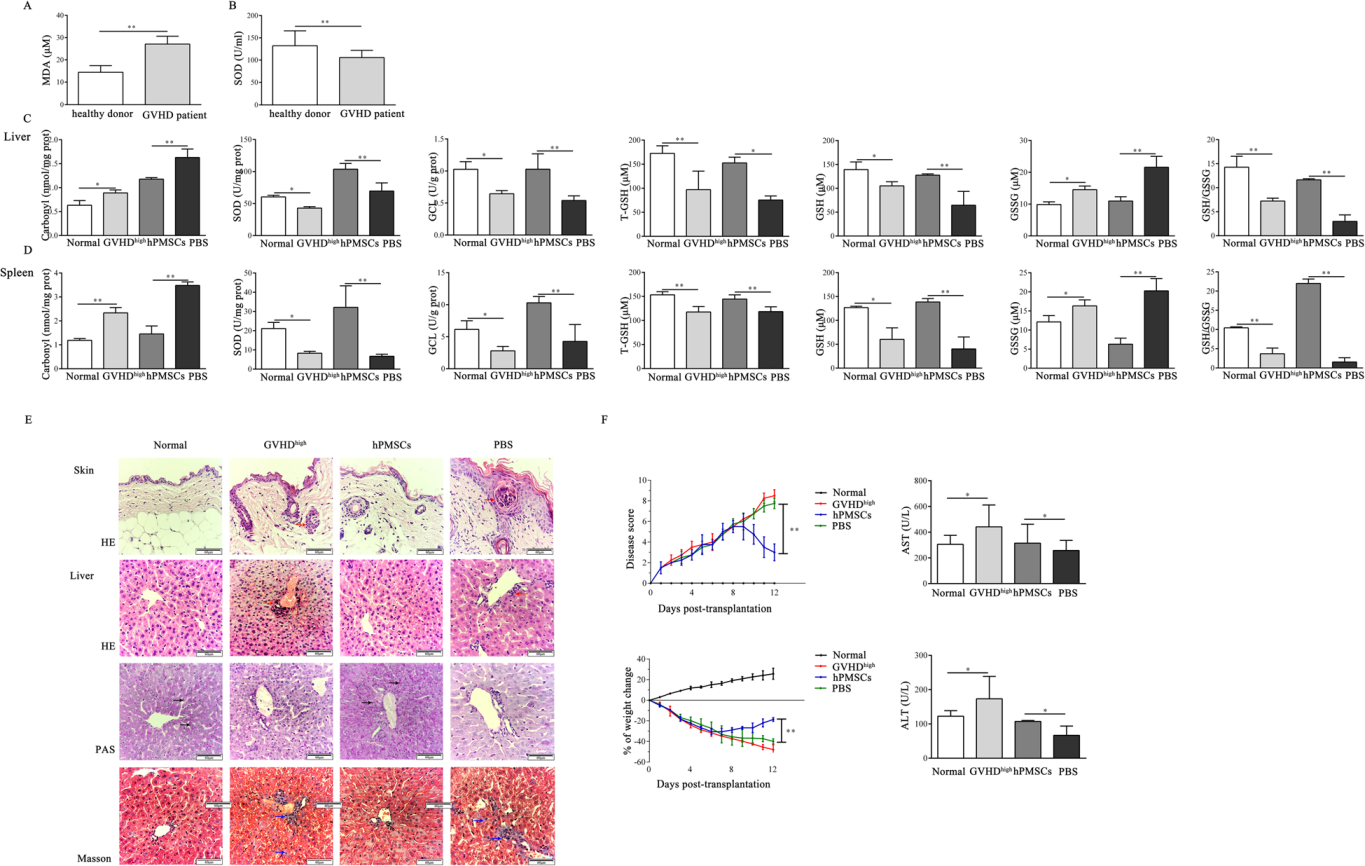
hPMSCs improve redox metabolism and alleviate GVHD symptoms. The level of MDA (**a**) and the activity of SOD (**b**) in GVHD patients and healthy donors (*n* = 7). The levels of carbonyl, T-GSH, GSH, and GSSG; the ratio of GSH/GSSG; and the activities of SOD and GCL in the spleen (**c**) and liver (**d**) of GVHD mice in different groups (*n* = 5). **e** Representative histological changes observed in the liver and skin samples harvested from different groups of GVHD mice. Scale bar = 60 μm. Masson’s trichrome and PAS staining analysis of the changes in the liver in different groups of GVHD mice, respectively. Red arrows indicate the infiltration of inflammatory cells, black arrows indicate the liver glycogen, and blue arrows indicate the collagen fibers. **f** Weight changes and disease scores and the activities of ALT and AST, in the different groups of GVHD mice (*n* = 5). The results were obtained from three independent experiments, *P < 0.05, **P < 0.01

Our previous research has shown the presence of an imbalance in redox metabolism during the development of GVHD [7, 10, 11]. To further explore the regulatory effects of hPMSCs on redox metabolism in GVHD, the MDA levels and SOD activity in the serum of GVHD patients and the levels of GSH and carbonyl and the activity of GCL and SOD were measured in the liver and spleen in the mouse model. The results showed that the MDA levels in the serum of GVHD patients were significantly increased; however, SOD activity was decreased compared to healthy donors (*P* < 0.01, Fig. 3a, b). Furthermore, the levels of carbonyl and GSSG were increased in the liver and spleen in the GVHD^high^ group compared to those in the normal group; however, the levels of total GSH (T-GSH) and GSH and the activities of SOD and GCL were decreased (liver: carbonyl, GSSG, GSH, SOD, and GCL: *P* < 0.05, T-GSH: *P* < 0.01; spleen: carbonyl and T-GSH: *P* < 0.01, GSSG, GSH, SOD, and GCL: *P* < 0.05; Fig. 3c, d), and the GSH/GSSG ratio in the GVHD^high^ group was lower than that in the normal group (*P* < 0.01, Fig. 3c, d). Furthermore, treatment with hPMSCs markedly decreased carbonyl and GSSG levels but increased the levels of T-GSH and GSH and the activities of SOD and GCL, compared to those in the PBS group (liver: carbonyl, GSSG, GSH, SOD, and GCL: *P* < 0.01, T-GSH: *P* < 0.05; spleen: *P* < 0.01; Fig. 3c, d). Similarly, the ratio of GSH/GSSG also increased after treatment with hPMSCs compared to that of the PBS group (*P* < 0.01, Fig. 3c, d).

Moreover, the H&E staining results showed remarkable evidence that treatment with hPMSCs improved the pathological changes in the liver and skin, such as leukocyte infiltration, fibrosis, and tissue damage, compared to those of the PBS group (Fig. 3e). Weights of mice used in the established model and disease scores were also monitored daily, and the results showed that weight loss was significantly improved and clinical scores were significantly decreased in the hPMSCs group compared to the PBS group (*P* < 0.01, Fig. 3f). Additionally, the Masson staining results in the liver showed that hPMSCs significantly reduced the fibrosis of liver tissue and bile duct area compared to those of the PBS group, and PAS staining results showed that the content of liver glycogen in the hPMSCs group increased compared to that in the PBS group (Fig. 3e). Additionally, as shown in Fig. 3f, the activities of alanine aminotransferase (ALT) and aspartate aminotransferase (AST) were decreased in the plasma of the GVHD^high^ group compared to the normal group; however, treatment with hPMSCs markedly increased the activities of ALT and AST in comparison with those of the PBS group (*P* < 0.05).

### hPMSCs enhance the expression of Nrf2 but inhibit the activation of NF-κB in the liver tissue of GVHD mice

To explore the regulatory effects of hPMSCs on the Nrf2 signaling pathway in GVHD, considering the existence of the crosstalk between Nrf2 and NF-κB, the levels of Nrf2 and downstream target genes (HO-1, NQO1, GCLC, and GCLM) and the phosphorylation levels of NF-κB and I-κB were determined in the liver and spleen tissues of the mouse model.

**Fig. 4:**
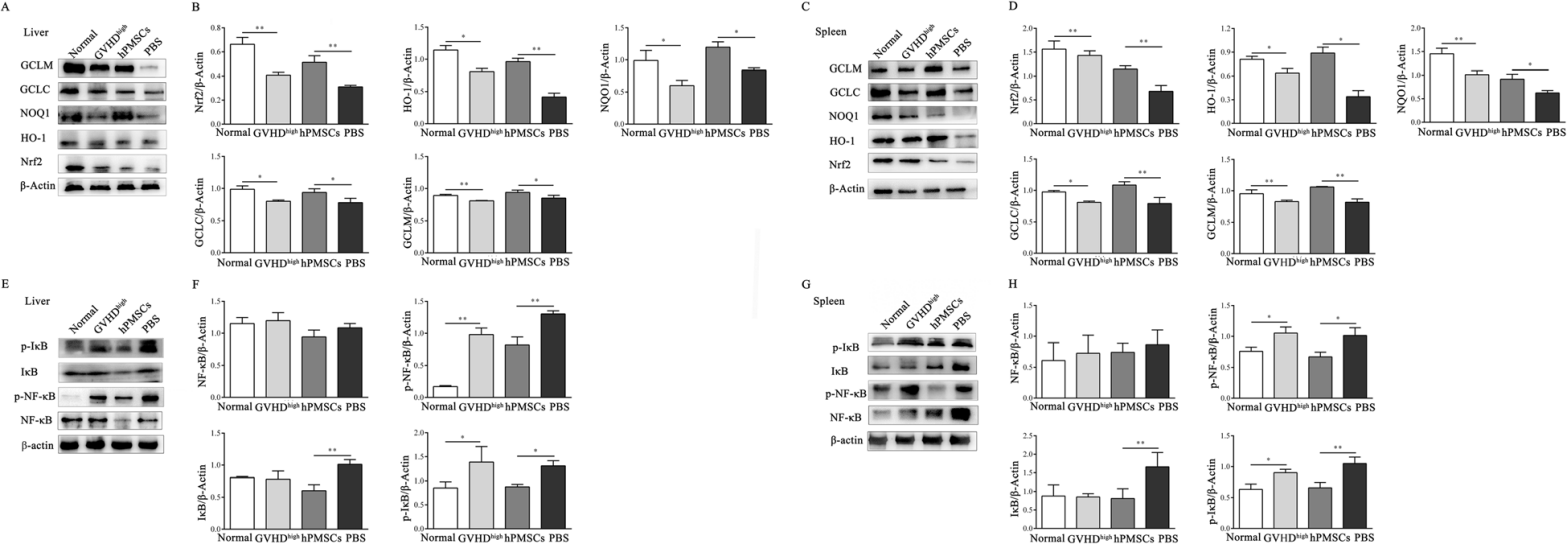
hPMSCs enhance Nrf2 expression but inhibit the activation of NF-κB in the GVHD mouse liver tissue. On day −5, Balb/c mice were conditioned using X-ray irradiation (8Gy); then, bone marrow cells and splenic mononuclear cells obtained from C57BL/6J mice were transplanted into the irradiated mice via injection into the tail vein. After 5 days, the mice demonstrated characteristics of GVHD at its peak (GVHD^high^). Concurrently, PBS and hPMSCs were used to treat the GVHD^high^ mice. The mice were then sacrificed to harvest the spleen and liver after treatment for 7 days for western blotting (WB). WB was performed to determine the expression of Nrf2, HO-1, NQO1, GCLC, and GCLM in the liver (**a**, **b**) and spleen (**c**, **d**) of GVHD mice in different groups (*n* = 5). WB was performed to determine the expression of phosphorylated NF-κB and I-κB in the liver (**e**, **f**) and spleen (**g**, **h**) of GVHD mice in different groups (*n* = 5). The results were obtained from three independent experiments *P <0.05, **P < 0.01

The WB results showed that the levels of Nrf2 and HO-1, NQO1, GCLC, and GCLM were decreased in the liver and spleen tissues in the GVHD^high^ group compared to those in the normal group (liver: Nrf2 and GCLM: *P* < 0.01, HO-1, NQO1, and GCLC: *P* < 0.05; spleen: Nrf2, HO-1, and GCLC: *P* < 0.05, NQO1 and GCLM: *P* < 0.01; Fig. 4a–d). However, after treatment with hPMSCs, Nrf2, HO-1, NQO1, GCLC, and GCLM levels were increased compared to those in the PBS group (liver: Nrf2 and HO-1: *P* < 0.01, NQO1, GCLC, and GCLM: *P* < 0.05; spleen: HO-1 and NQO1: *P* < 0.05, Nrf2, GCLM, and GCLC: *P* < 0.01; Fig. 4a–d).

As shown in Fig. 4e–h, the levels of p-NF-κB and p-I-κB were increased in the liver and spleen tissue in the GVHD^high^ group compared to those in the normal group (liver: p-NF-κB: *P* < 0.01, p-I-κB: *P* < 0.05; spleen: *P* < 0.05). The treatment with hPMSCs significantly decreased the levels of p-NF-κB and p-I-κB compared to those in the PBS group (liver: p-NF-κB: *P* < 0.01, p-I-κB: *P* < 0.05; spleen: p-NF-κB: *P* < 0.05, p-I-κB: *P* < 0.01; Fig. 4e–h).

### hPMSCs enhance the expression of Nrf2 but inhibit the activation of NF-κB in mononuclear cells of GVHD mice

**Fig. 5:**
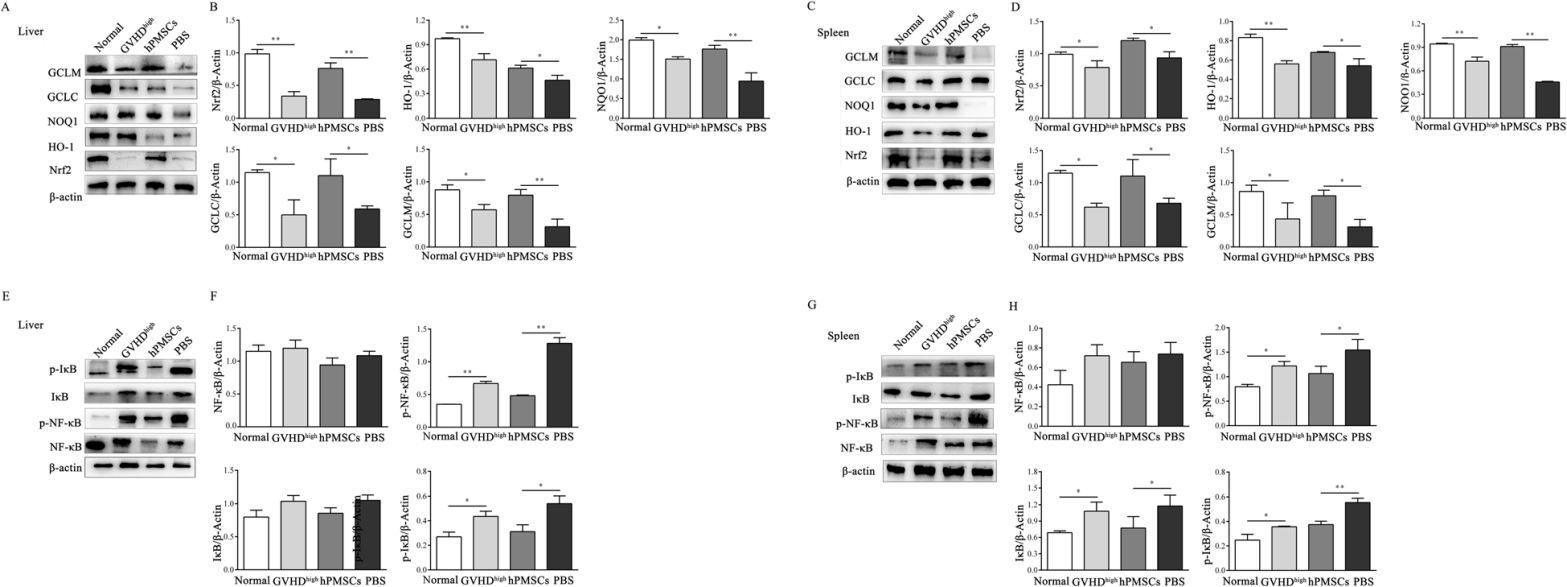
hPMSCs enhance Nrf2 levels but inhibit the activation of NF-κB in the mononuclear cells of GVHD mice. The mice were sacrificed to harvest the mononuclear cells of the spleen and liver after the treatment for 7 days for WB. WB was performed to determine the expression of Nrf2, HO-1, NQO1, GCLC, and GCLM in the mononuclear cells of the liver (**a**, **b**) and spleen (**c**, **d**) of GVHD mice in different groups (*n* = 5). WB was performed to determine the expression of phosphorylated NF-κB and I-κB in the mononuclear cells of the liver (**e**, **f**) and spleen (**g**, **h**) of GVHD mice in different groups (*n* = 5). The results were obtained from three independent experiments, *P < 0.05, **P < 0.01

Considering that hPMSCs can alleviate the inflammatory response elicited in GVHD by regulating the GSH and GST levels in T cells [7], based on the above-mentioned changes of Nrf2 and NF-κB levels in tissues, the Nrf2 and NF-κB levels in mononuclear cells in the liver and spleen tissues of the mouse model were also analyzed. As illustrated in Fig. 5, the Nrf2, HO-1, NQO1, GCLC, and GCLM levels were decreased in liver and spleen mononuclear cells in the GVHD^high^ group compared to those in the normal group (liver: Nrf2 and HO-1: *P* < 0.01, NQO1, GCLC, and GCLM: *P* < 0.05; spleen: HO-1, NQO1: *P* < 0.01, Nrf2, GCLC, and GCLM: *P* < 0.01; Fig. 5a–d). However, the levels of Nrf2, HO-1, NQO1, GCLC, and GCLM in mononuclear cells were increased after hPMSC intervention compared to those in the PBS group (liver: HO-1 and GCLC: *P* < 0.05, Nrf2, NQO1, and GCLM: *P* < 0.01; spleen: Nrf2, HO-1, GCLM, and GCLC: *P* < 0.05, NQO1: *P* < 0.01; Fig. 5a–d). In contrast, the levels of p-NF-κB and p-I-κB were increased in liver and spleen mononuclear cells in the GVHD^high^ group compared to those in the normal group (liver: p-NF-κB: *P* < 0.01, p-I-κB: *P* < 0.05; spleen: *P* < 0.05, Fig. 5e–h). The p-NF-κB and p-I-κB levels in mononuclear cells were decreased after hPMSC intervention compared to those in the PBS group (liver: p-NF-κB: *P* < 0.01, p-I-κB: *P* < 0.05; spleen: p-NF-κB: *P* < 0.05, p-I-κB: *P* < 0.01; Fig. 5e–h).

### hPMSCs induce the differentiation of CD4^+^IL-10^+^ T cells by controlling the activation of the Nrf2 and NF-κB signaling pathways

**Fig. 6:**
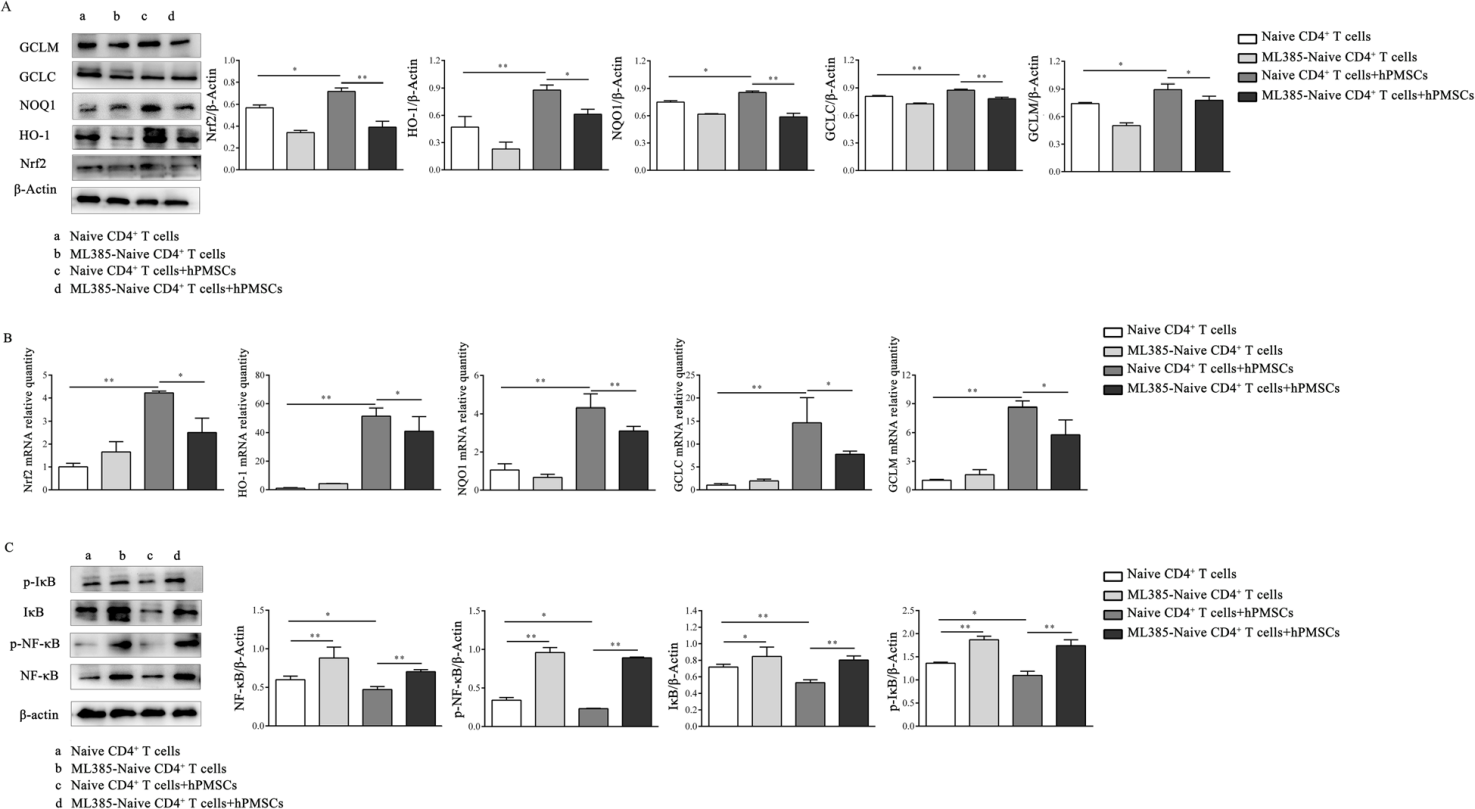
hPMSCs induce the differentiation of CD4^+^IL-10^+^ T cells by controlling the activation of the Nrf2 and NF-κB pathways. CD4^+^IL-10^+^ T cells were induced, accompanied by changes in IL-2, IL-10, IL-27, and IFN-α2b levels in vitro, by using the naive CD4^+^ T cells treated with or without the Nrf2 inhibitor ML385 for 12 h, and then naive CD4^+^ T cells were cultured with or without hPMSCs and collected after 3 days for WB. **a** WB was performed to determine the expression of Nrf2, HO-1, NQO1, GCLC, and GCLM in the different CD4^+^IL-10^+^ T cell differentiation systems in vitro. **b** qRT-PCR analysis of the expression of Nrf2, HO-1, NQO1, GCLC, and GCLM in the different CD4^+^IL-10^+^ T cell differentiation systems in vitro. **c** WB was performed to determine the expression of phosphorylated NF-κB and I-κB in the different CD4^+^IL-10^+^ T cell differentiation systems in vitro. The results were obtained from three independent experiments, *P < 0.05, **P < 0.01

To further confirm the role of the Nrf2 signaling pathway in the generation of CD4^+^IL-10^+^ T cells, expression of Nrf2, its downstream region, and expression of NF-κB were also analyzed during the formation of CD4^+^IL-10^+^ T cells from naive CD4^+^T cells. As demonstrated by the results obtained, when hPMSCs were present, the levels of Nrf2, HO-1, NQO1, GCLC, and GCLM were significantly increased compared to those in the hPMSC-free group (WB: Nrf2, NQO1, and GCLM: *P* < 0.05, HO-1 and GCLC: *P* < 0.01; RT-PCR: *P* < 0.01; Fig. 6a, b). After naive CD4^+^ T cells pretreated with ML385 were cocultured with hPMSCs, the levels of Nrf2, HO-1, NQO1, GCLC, and GCLM were decreased significantly compared to those of the untreated naive CD4^+^ T cells cocultured with hPMSCs (WB: Nrf2, NQO1, and GCLC: *P* < 0.01, HO-1 and GCLM: *P* < 0.05; RT-PCR: NQO1: *P* < 0.01, Nrf2, HO-1, GCLC, and GCLM: *P* < 0.05; Fig. 6a, b). Moreover, the results showed that when hPMSCs were present, the levels of p-NF-κB and p-I-κB were significantly decreased compared to those in the group where hPMSCs were absent (*P* < 0.01, Fig. 6c). In the presence of hPMSCs, the levels of p-NF-κB and p-I-κB increased significantly in ML385-pretreated naive CD4^+^ T cells compared to those observed in the untreated naive CD4^+^ T cells (*P* < 0.01, Fig. 6c).

### hPMSCs upregulate the expression of Nrf2 by inhibiting the levels of NF-κB in the nucleus of CD4^+^IL-10^+^ T cells

**Fig. 7:**
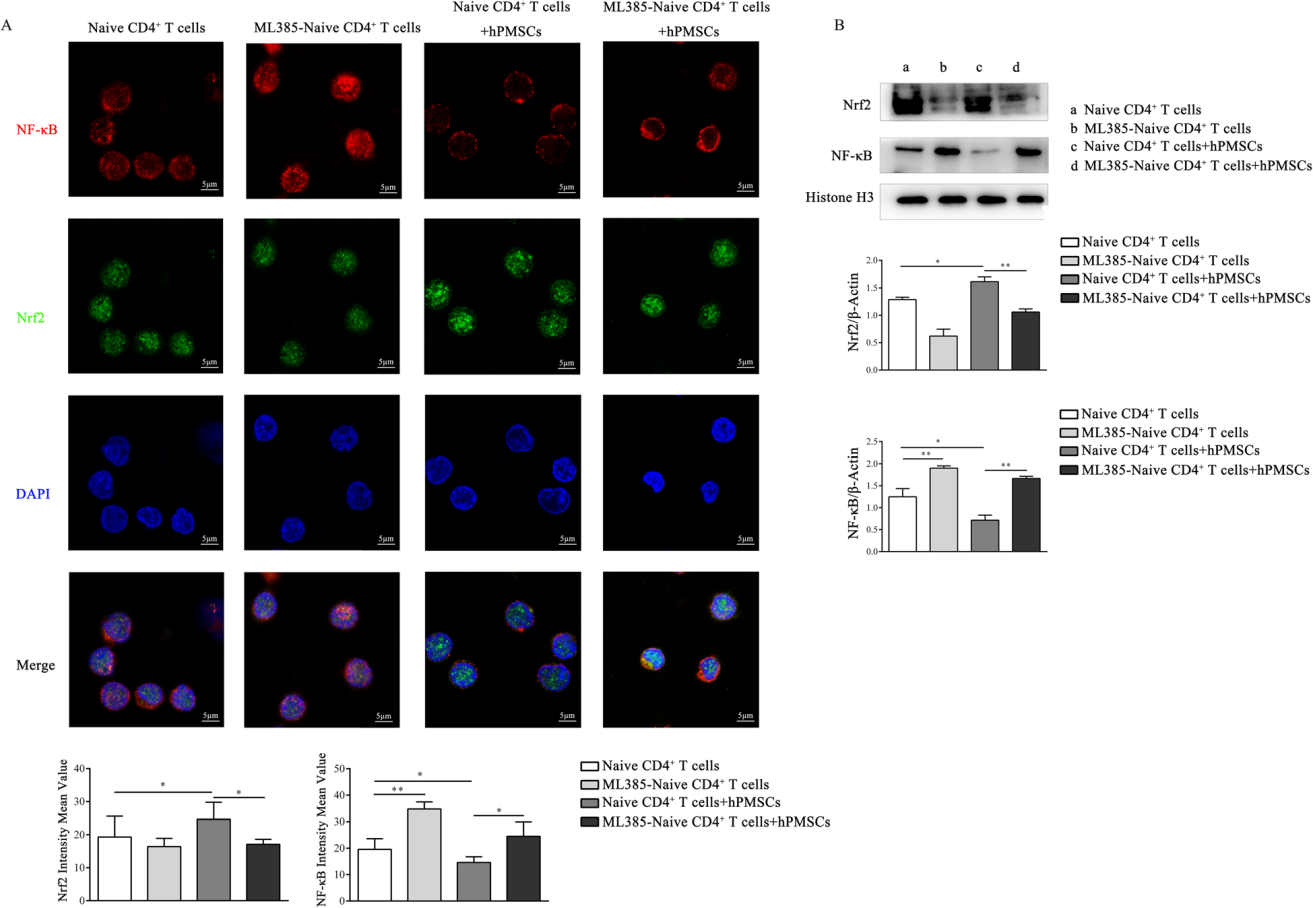
hPMSCs upregulate Nrf2 expression by inhibiting NF-κB levels in the nucleus of CD4^+^IL-10^+^ T cells. CD4^+^IL-10^+^ T cells were induced, using the naive CD4^+^ T cells treated with or without the Nrf2 inhibitor ML385 for 12 h, and then naive CD4^+^ T cells were cultured with or without hPMSCs. The cells and the nuclear and cytoplasmic proteins were collected for analysis after 3 days. **a** Immunofluorescence (IF) assays were performed to determine the expression of Nrf2 and NF-κB in the nucleus of CD4^+^IL-10^+^ T cells in the differentiation system in a different group in vitro. **b** WB was performed to determine the expression of Nrf2 and NF-κB in the nucleus of CD4^+^IL-10^+^ T cells in the differentiation system in a different group in vitro. The results were obtained from three independent experiments, *P < 0.05, **P < 0.01

Previous studies have indicated that Nrf2 and NF-κB can competitively translocate into the nucleus [22]; thus, the levels of Nrf2 and NF-κB in the nucleus of CD4^+^IL-10^+^T cells were further determined during the formation of CD4^+^IL-10^+^ T cells from naive CD4^+^ T cells. As shown in Fig. 7a and b, the Nrf2 levels in the nucleus of CD4^+^IL-10^+^ T cells were higher in the presence of hPMSCs than those observed in the absence of hPMSCs (*P* < 0.05), but the levels of NF-κB were lower (IF: *P* < 0.05; WB: *P* < 0.01). Further analysis showed that when ML385-pretreated naive CD4^+^ T cells were cocultured with hPMSCs, the Nrf2 levels in the nucleus of CD4^+^IL-10^+^ T cells markedly decreased (IF: *P* < 0.05; WB: *P* < 0.01), while the levels of NF-κB increased compared to those observed in untreated naive CD4^+^ T cells cocultured with hPMSCs (IF: *P* < 0.05; WB: *P* < 0.01).

## Discussion

The application of MSCs has become a promising new strategy for the treatment of inflammation-related diseases, such as GVHD, due to the immunosuppressive ability demonstrated by these cells. However, the mechanism by which MSCs simultaneously exert their antioxidant and immunosuppressive effects for the treatment of GVHD remains elusive. When GVHD occurs, high levels of ROS in tissues and low levels of GSH and antioxidant components are observed, which are the predominant reasons for infliction of damage to the target organs, such as the liver. In this study, we observed that the levels of MDA, an index of ROS-mediated lipid peroxidation, were increased, and the activity of SOD, an enzyme that plays an antioxidant role by scavenging ROS, decreased in the serum samples obtained from GVHD patients. We further confirmed the changes in redox metabolism during GVHD development and found that the activities of SOD and GCL and the ratio of GSH/GSSG in the liver and spleen in the allogeneic GVHD mouse model were also significantly reduced, but the levels of carbonyl, an index of protein peroxidation, increased. These results suggest that there exists an imbalance in redox metabolism during GVHD development. Interestingly, after treatment with hPMSCs, the levels of carbonyl decreased, while the activities of SOD and GCL and GSH/GSSG ratios increased in the GVHD mouse model, which suggested that hPMSCs could ameliorate the imbalance in oxidation and antioxidant metabolism in GVHD. Additionally, after treatment with hPMSCs, lymphocyte infiltration and damage to the liver and skin were alleviated, body weights and disease scores were improved, and activities of ALT and AST tended to shift more towards those observed in the normal group. These results indicate that hPMSCs can improve GVHD by maintaining a balance of redox metabolism.

PD-1 was first discovered and considered as a molecule that could function along with PD-L1 and induce the apoptosis of activated T cells [23, 24]. Our previous study has shown that the levels of PD-1 in activated CD4^+^ T cells increased in a GVHD mouse model and were associated with high levels of ROS during the development of GVHD. The hPMSCs can attenuate the levels of ROS and PD-1 by enhancing the levels of GSH and GST in CD4^+^ T cells [7]. Additionally, elevated PD-1 expression on the surface of T cells was detected in both EAE and rheumatoid arthritis (RA) mouse models [25]. CD4^+^IL-10^+^ T cells reportedly contribute to hPMSC-mediated regulation of the inflammatory response [6]. In this study, we found that the proportion of CD4^+^IL-10^+^ T cells decreased in GVHD patients and the GVHD mouse model, while the PD-1 expression levels in CD4^+^IL-10^+^ T cells markedly increased, which suggested that PD-1 expression was associated with the percentage of CD4^+^IL-10^+^ T cells and the development of GVHD. Interestingly, we further observed that hPMSCs could inhibit the expression of PD-1 in CD4^+^IL-10^+^ T cells and protect CD4^+^IL-10^+^ T cells from apoptosis. These findings remarkably suggest that one of the most important mechanisms by which hPMSCs exert anti-inflammatory effects may rely on increasing the percentage of CD4^+^IL-10^+^ T cells realized through downregulation of expression of PD-1.

Evidently, activation of the NF-κB signaling pathway can promote elicitation of the inflammatory response and trigger the occurrence of tissue damage [26]. Nrf2, a key transcription factor, can enter the nucleus to mediate the transcription of endogenous antioxidant genes, such as NQO1 and HO-1, under stress conditions and increase the levels of corresponding antioxidant proteins, thus mitigating oxidative damage. However, it has been reported that NF-κB can bind to CREB-binding protein and compete with Nrf2 in the nucleus, thereby inhibiting activation of the Nrf2 pathway [27]. In this study, we found that hPMSCs could significantly improve the levels of Nrf2 and antioxidant components, such as HO-1, NQO1, GCLC, and GCLM, and inhibit the phosphorylation of NF-κB in the liver tissue. These findings suggest that hPMSCs can reduce the oxidative stress response and further mitigate liver injury by regulating the crosstalk between Nrf2 and NF-κB. Consistent with our findings, Lim et al. have shown that GSH synthesis and redox cycling, which are mediated through the Nrf2 signaling pathway, are important cellular factors that mediate the therapeutic functions of hES-MSCs for GVHD treatment [28]. The inflammatory response triggered by the oxidative stress of mononuclear cells is another major cause of target organ damage observed in GVHD; thus, in this study, we further analyzed the regulation effects of hPMSCs on the Nrf2 and NF-κB pathways in mononuclear cells in the liver and spleen of the GVHD mouse model. We found that hPMSCs could also inhibit the phosphorylation of NF-κB and enhance the expression of Nrf2, HO-1, NQO1, GCLC, and GCLM. These results indicate that hPMSCs may regulate the activation of the Nrf2 and NF-κB signaling pathways in mononuclear cells, thereby reducing the intensity of the inflammation response.

In a previous study, we found that PD-1 expression in CD4^+^T cells was related to the ROS levels observed in a GVHD mouse model. Here, the higher expression of PD-1 in CD4^+^IL-10^+^ T cells was observed in both GVHD patients and the chosen mouse model. Therefore, we hypothesized that hPMSCs could alleviate GVHD by regulating the expression of PD-1 in CD4^+^IL-10^+^ T cells by controlling the crosstalk between Nrf2 and the NF-κB signaling pathway. A CD4^+^IL-10^+^ T cell differentiation model with or without Nrf2 inhibitor-ML385 was established in vitro, and the PD-1 expression in CD4^+^IL-10^+^ T cells affected by hPMSCs was detected. The results showed that PD-1 levels decreased after coculturing with hPMSCs and the ability of hPMSCs to inhibit the expression of PD-1 was partially reversed in the presence of ML385, which suggested Nrf2 was involved in the hPMSC-mediated regulation of the expression of PD-1 in CD4^+^IL-10^+^ T cells. In addition to the findings showing that the expression of PD-1 was positively correlated with apoptosis of CD4^+^IL-10^+^ T cells, we suggested that hPMSCs increased the percentages of CD4^+^IL-10^+^ T cells by inhibiting PD-1-induced apoptosis through regulation of the Nrf2 pathways. However, the detailed mechanism warrants further exploration.

The relationship between the generation of CD4^+^IL-10^+^ T cells induced by hPMSCs and the Nrf2 and NF-κB signaling pathways was analyzed. The results showed that in the presence of hPMSCs, the phosphorylation of NF-κB was decreased and the inhibitory effects of hPMSCs on the phosphorylation of NF-κB were reduced during ML385-pretreated naive CD4^+^ T cell differentiation into CD4^+^IL-10^+^ T cells. Interestingly, the increased effects of hPMSCs on Nrf2 and HO-1 expression, which was related to the expression of IL-10 [18], were partly inhibited. Consistent with these results, the immunofluorescence results showed that hPMSCs could promote Nrf2 nuclear translocation and inhibit NF-κB nuclear translocation during the differentiation of CD4^+^IL-10^+^ T cells. Thus, the crosstalk between Nrf2 and NF-κB seems to be involved in the generation of CD4^+^IL-10^+^ T cells. The presence of hPMSCs could inhibit the expression levels of PD-1 and NF-κB and, in turn, could promote the generation of CD4^+^IL-10^+^ T cells.

**Fig. 8:**
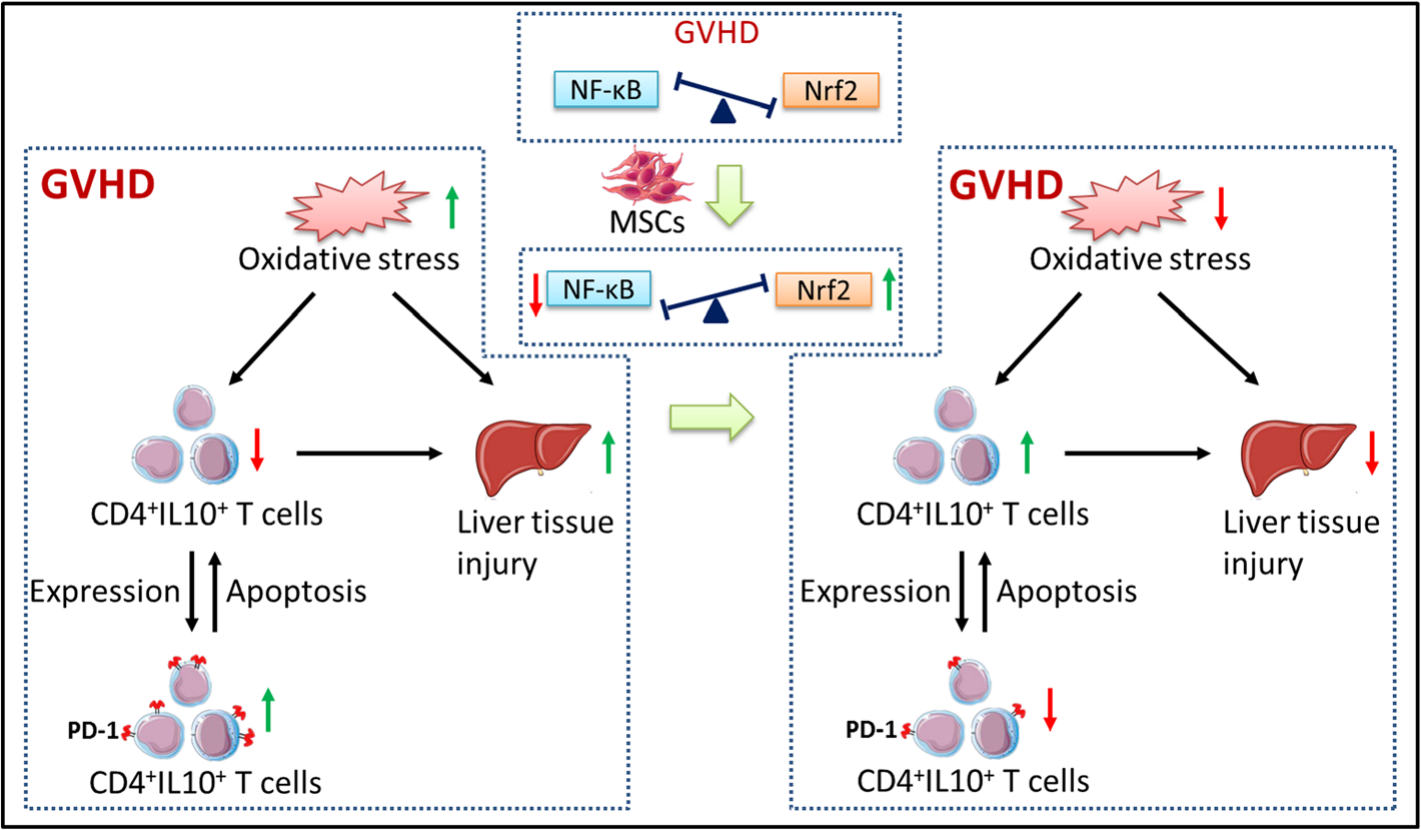
Summary of hPMSC-mediated PD-1 expression on CD4^+^IL-10^+^ T cells and mitigation of liver damage in GVHD model mice. The oxidative stress aggravated during GVHD and indirectly induced by the imbalance in the percentage of CD4^+^IL-10^+^T cells and the expression of PD-1 on CD4^+^IL-10^+^T cells. These factors elicited the inflammatory and redox response in the GVHD liver and resulted in the occurrence of liver damage. The hPMSCs can enhance Nrf2 activation and attenuate the activation of NF-κB, which reduces the oxidative stress and increase the percentage of CD4^+^IL-10^+^T cells by suppressing the CD4^+^IL-10^+^T cell apoptosis induced by PD-1 in CD4^+^IL-10^+^T cells. Both effects reduce the inflammatory and redox responses, further alleviating the liver damage observed in GVHD mice

In summary, as shown in Fig. 8, we demonstrated that redox metabolism was imbalanced and PD-1 expression increased in CD4^+^IL-10^+^ T cells by analyzing GVHD clinical samples and mouse models. The hPMSCs can mitigate the pathological damage in GVHD mouse model liver tissues by maintaining the balance in redox metabolism via enhancement of Nrf2 activation and attenuation of the activation of NF-κB in both tissues and mononuclear cells. However, hPMSCs downregulated the expression of PD-1 in CD4^+^IL-10^+^ T cells by regulating the crosstalk between the Nrf2 and NF-κB signaling pathways, which inhibited CD4^+^IL-10^+^ T cell apoptosis, thus promoting the generation of CD4^+^IL-10^+^ T cells and further mitigating the inflammatory response of GVHD.

## Conclusions

We have showed that hPMSCs could mitigate the hepatic pathological damage in the GVHD mouse model by maintaining the balance in redox metabolism in tissues, as well as mononuclear cells, and CD4^+^IL-10^+^ T cells, which were associated with the enhancement of Nrf2 activation and attenuation of NF-κB activation and the inhibition of PD-1 expression in CD4^+^IL-10^+^ T cells. Therefore, these findings demonstrated the effectiveness of hPMSCs in the treatment of GVHD based on the aspects of antioxidation and immunomodulation and provided evidence for the potential use of hPMSCs in clinical cell therapies.

## Data Availability

All datasets in this article are included within the article and additional files.

## Abbreviations

GVHD: Graft-versus-host disease
hPMSCs: Human placenta-derived mesenchymal stromal cells
allo-HSCT: Allogeneic hematopoietic stem cell transplantation
PD-1: Programmed death-1
GSH: Glutathione
SOD: Superoxide dismutase
Nrf2: Nuclear factor-E2-related factor 2
NQO1: NAD (P) H: quinone oxidoreductase 1
HO-1: Heme oxygenase 1
GCLM: Glutamate-cysteine ligase regulatory subunit
GCLC: Glutamate-cysteine ligase catalytic subunit
NF-κB: Nuclear factor kappa-B
PBMCs: Peripheral blood mononuclear cells
ALT: Alanine aminotransferase
AST: Aspartate aminotransferase
